# Development and evaluation of single-plex TaqMan real-time quantitative PCR assays for the detection of six tick-borne pathogenic viruses in northeastern China

**DOI:** 10.3389/fcimb.2026.1777543

**Published:** 2026-06-04

**Authors:** Shiyao Xu, Zheng Gui, Ziyan Liu, Kai Wang, Ning Liu, Yunzhi Peng, Liang Li, Zedong Wang

**Affiliations:** 1 Department of Infectious Diseases, Center of Infectious Diseases and Pathogen Biology, State Key Laboratory of Zoonotic Diseases, the First Hospital of Jilin University, Changchun, Jilin, China; 2 College of Life Science, Northeast Forestry University, Harbin, Heilongjiang, China; 3 State Key Laboratory of Pathogen and Biosecurity, Changchun Veterinary Research Institute, Chinese Academy of Agricultural Sciences, Changchun, Jilin, China; 4 International Center of Future Science, Jilin University, Changchun, Jilin, China

**Keywords:** northeastern China, real-time quantitative PCR (RT-qPCR), tick-borne diseases, tick-borne viruses, ticks

## Abstract

**Background:**

Northeastern China is a recognized hotspot for tick-borne viral diseases. However, there remains a lack of methods capable of simultaneously detecting these emerging and re-emerging tick-borne viruses.

**Methods:**

Primer and probe sets were designed using Beacon Designer 8.0, targeting conserved regions of viral genomes from Alongshan virus (ALSV), Tick-borne encephalitis virus (TBEV), Severe fever with thrombocytopenia syndrome virus (SFTSV), Beiji nairovirus (BJNV), Yezo virus (YEZV), and Songling virus (SGLV). Recombinant plasmids were constructed to assess assay sensitivity, while virus-positive cDNA from tick samples was used to verify specificity. The performance of these assays was further validated using field-collected tick samples, with results compared against established reference methods serving as gold standards.

**Results:**

Specific primer and probe sets were designed with amplicon lengths ranging from 83 to 199 bp. Optimization of the reaction components yielded final primer volumes of 0.2–0.5 μL and probe volumes of 0.4–1.0 μL (from 10 μM working stocks). Sensitivity analysis demonstrated a limit of detection (LOD) as low as 10 copies/μL for all six viruses, while specificity testing confirmed no cross-reactivity among the targets. In validation trials, the established TaqMan RT-qPCR assays showed complete concordance with reference methods for ALSV (5/15) and TBEV (4/14). Notably, the TaqMan assays identified additional positive samples for SFTSV (n=1), BJNV (n=3), and YEZV (n=1) that were missed by the reference assays, particularly in samples with low viral loads. Conversely, two samples that tested positive for SGLV using SYBR Green RT-qPCR yielded negative results with the TaqMan RT-qPCR assay.

**Conclusion:**

We have successfully developed a suite of highly sensitive and specific single-plex TaqMan RT-qPCR assays for the rapid detection of six key tick-borne viruses in northeastern China. These assays facilitate efficient viral surveillance in tick populations and provide a diagnostic tool with significant potential for clinical applications.

## 1 Introduction

Tick-borne diseases represent a growing threat to global public health, particularly due to the risks associated with tick-borne viral infections (Zhou et al., 2023). Recent advancements in next-generation sequencing technology have facilitated the identification of thousands of tick-borne viruses worldwide, many of which are capable of human infection (Shi et al., 2016; Ni et al., 2023; Zhou et al., 2023). The increasing diversity of these pathogens poses a significant challenge for the effective diagnosis, prevention, and control of tick-borne diseases on a global scale.

Northeastern China is a recognized hotspot for tick-borne viral diseases (Liu and Wang, 2025). To date, ten pathogenic tick-borne viruses have been identified in this region, primarily belonging to the families *Flaviviridae*, *Nairoviridae*, and *Phenuiviridae*. However, there remains a lack of methods capable of simultaneously detecting these tick-borne viruses. In this study, single-plex TaqMan RT-qPCR assays were developed for six human pathogenic tick-borne viruses previously identified in northeastern China: Alongshan virus (ALSV), Tick-borne encephalitis virus (TBEV), Severe fever with thrombocytopenia syndrome virus (SFTSV), Beiji nairovirus (BJNV), Yezo virus (YEZV), and Songling virus (SGLV).

Among these viruses, TBEV is a single-stranded, positive-sense RNA flavivirus that can cause fatal encephalitis, while ALSV is a novel four-segmented flavivirus associated with human febrile illness (Sun et al., 2017; Gomer et al., 2024). SGLV, YEZV, and BJNV are emerging members of the *Nairoviridae* family; specifically, SGLV and YEZV are three-segmented, negative-strand RNA viruses, whereas BJNV is a two-segmented, negative-strand RNA virus, all of which cause febrile illnesses lacking specific clinical features (Kodama et al., 2021; Ma et al., 2021; Wang et al., 2021). SFTSV is a three-segmented, negative-strand RNA phenuivirus that causes a severe febrile illness characterized by profound thrombocytopenia, leukopenia, and a high risk of multi-organ failure (Zhang et al., 2021; Kim and Park, 2023). The establishment of detection methods for these six representative tick-borne viruses will facilitate the differential diagnosis of tick-borne diseases in northeastern China.

## 2 Materials and methods

### 2.1 Viral samples, ticks, and RNA extraction

Positive cDNA samples for ALSV, TBEV, SFTSV, BJNV, YEZV, and SGLV were obtained from ticks in our previous studies and stored in our laboratory at -80 °C (Liu et al., 2016, 2022; Lv et al., 2023). Questing ticks were collected via the flagging method in northeastern China from May 2020 to June 2023. Tick species were identified using morphological and molecular methods as describe elsewhere (Shao et al., 2020). Ticks were pooled (n=10) by species and site, then homogenized via Tissuelyser (70 Hz, 2 min). Lysates were centrifuged (12, 000 rpm, 10 min, 4 °C) to obtain supernatants and pooled for RNA library construction and meta-transcriptomic sequencing. Based on the transcriptomic results, samples in the pooled libraries containing ALSV, TBEV, SFTSV, BJNV, YEZV, and SGLV were selected as field samples for downstream assay validation.

Viral RNA was extracted from tick samples using the TIANamp Virus RNA Kit (TIANGEN, China) and reverse transcribed into complementary DNA (cDNA) using PrimeScript RT Master Mix (TaKaRa, Japan), according to the manufacturers’ instructions. The resulting cDNA was stored at -80 °C until further use.

### 2.2 Primer design and concentration optimization

Complete genome sequences of ALSV, TBEV, SFTSV, BJNV, YEZV, and SGLV strains identified in northeastern China and the Russian Far East were downloaded from the GenBank of NCBI (**Supplementary Table 1**). The conserved regions of these viruses were analyzed to design specific primers and probes using Beacon Design 8.0 software.

An assay was conducted using the Accurate 96-x6 Real-Time PCR Detection System (Dragonlab, China) to determine the optimal reaction conditions, including primer and probe concentrations and annealing temperature. Various primer volumes (final concentrations of 0.05–0.25 μM) and probe volumes (final concentrations of 0.1–0.5 μM), along with annealing temperatures of 56, 57, 58, 59, and 60 °C, were tested to identify the optimal conditions. The reaction system exhibiting the lowest Ct value and highest fluorescence intensity was determined to be optimal.

### 2.3 Reaction system and conditions

All single-plex TaqMan RT-qPCR assays were performed in 20 μL reaction volumes. Each reaction mixture consisted of 12.5 μL Premix Ex Taq, 0.2 to 0.5 μL of each primer, 0.4 to 1.0 μL of probe, 1 μL of plasmid template or cDNA derived from tick samples, and sterile ddH_2_O to a final volume of 20 μL, with nuclease-free ddH_2_O and cDNA derived from lab-reared pathogen-free tick colonies serving as the negative control. Amplification was conducted on the Accurate 96-x6 Real-Time PCR Detection System (Dragonlab, China) under the following thermal cycling conditions: initial predenaturation at 95 °C for 30 s, followed by 50 cycles of denaturation at 95 °C for 5 s and annealing at 60 °C for 30 s.

### 2.4 Plasmid standards and standard curve preparation

The fragments for plasmid construction of the six viruses were amplified by semi-nested PCR and purified using the PCR Purification Kit (TaKaRa, Japan) following the manufacturer’s instructions. Purified DNA fragments were cloned into the pMD18-T Vector (TaKaRa, Japan) and transformed into Escherichia coli DH5α competent cells (Invitrogen, USA). Positive clones were selected by blue/white screening and further validated through colony PCR and Sanger sequencing. Plasmids were extracted using the TIANprep Mini Plasmid Kit (TIANGEN, China), and concentrations were determined using a NanoDrop 2000 spectrophotometer (ThermoFisher, USA). Plasmid copy numbers were calculated using the formula: copies/μL = [6.02 × 10²³ × DNA concentration (g/μL)]/[average molecular weight per base pair (g/mol) × template length (bp)]. The plasmids were then diluted to 1 × 10^10^ copies/μL and stored at -20 °C for downstream applications. The standard plasmid constructs, with final reaction concentrations of 10^8^ to 10^1^ copies/µL, was used as a template for amplification to generate the standard curves of the RT-qPCR assays.

### 2.5 Sensitivity and specificity analysis

To evaluate the sensitivity of the established RT-qPCR assays, the standard plasmids were of the six viruses were serially tenfold diluted in sterile ddH_2_O, with concentrations ranging from 10^8^ to 10^-^¹ copies/μL. These diluted plasmids were subsequently used as templates in RT-qPCR amplification. To assess potential cross-reactivity among the RT-qPCR assays for pathogenic tick-borne viruses, ALSV-, TBEV-, SFTSV-, BJNV-, YEZV-, and SGLV- positive tick cDNA samples were employed. Nuclease-free water served as the negative control template.

### 2.6 Detection of the viruses in field samples

In total, target viruses were confirmed via meta-transcriptomic sequencing in multiple RNA libraries constructed from questing ticks collected in northeastern China: 55 pools of *Ixodes persulcatus* (comprising 17, 14, 17, and 7 pools), 11 pools of *Haemaphysalis longicornis*, and 9 pools of *H. concinna* (**Supplementary Table 2**). These tick pools were further verified using reference assays from previous studies, including semi-nested PCR for detecting ALSV (Wang et al., 2019), TBEV (Li et al., 2022), SFTSV (Kim et al., 2021), and BJNV (Wang et al., 2021), as well as the TaqMan RT-qPCR assay for YEZV (Kodama et al., 2021) and the SYBR Green RT-qPCR assay for SGLV (Ma et al., 2021). These assays were employed as gold standard methods for analyzing the viruses in the tick samples. Then samples were detected using the established ALSV-, TBEV-, SFTSV-, BJNV-, YEZV-, and SGLV- specific RT-qPCR assays, respectively. Nuclease-free water and cDNA from laboratory-reared, pathogen-free tick colonies were used as negative controls in place of field tick cDNA templates.

## 3 Results

### 3.1 Primer design and concentration optimization

The specific primers and probes for the six viruses are listed in **Table 1**, with amplified fragment lengths of 180 bp for ALSV, 160 bp for TBEV, 199 bp for SFTSV, 83 bp for BJNV, 134 bp for YEZV, and 172 bp for SGLV. The optimal primer volumes for the viruses ranged from 0.2 to 0.5 μL, and probe volumes ranged from 0.4 to 1.0 μL (The working concentrations of primers and probes were 10 μM) (**Table 1**), with an optimal annealing temperature of 60 °C.

**Table 1:** Primers and probes used for the detection of six tick-borne viruses circulating in northeastern China.

Virus	Primer and probe sequences (5’-3’)	Volume (µL)	Target genes	Product length (bp)
ALSV-F	GCTTGTGGTCATCATYATG	0.2	VP1b	180
ALSV-R	CTCTGCCACATRCTGAWG	0.2		
ALSV-P	FAM-CTCTCGTCRGCYATACCRCCA-BHQ1	0.4		
TBEV-F	CAYGCAAAGCTRTCGGATACCA	0.2	E	160
TBEV-R	YGGTGYTCTTCAGCCARRGT	0.2		
TBEV-P	FAM-TYGCGGCCAGATGCCCHACAA-BHQ1	0.4		
SFTSV-F	GAGTCTAGGTCATCTGATC	0.2	RdRP	199
SFTSV-R	CYGTCTCTGTCTTTATGTAAG	0.2		
SFTSV-P	FAM-AGCAATGACAGAYGCCTTCCA-BHQ1	0.4		
BJNV-F	CAGAGYCCRCAAGGTAARAAR	0.5	NP	83
BJNV-R	GGTSGATTTSTGTGSTCTTTG	0.5		
BJNV-P	FAM-ATGCTRCAACAGATGGGYAGYCCAA-BHQ1	1		
YEZV-F	YACRGARGAGCARGAACAAGA	0.3	NP	134
YEZV-R	CAYGAAGGCAGGCTRAAGA	0.3		
YEZV-P	FAM-ACTGGGTGAAAGGAGGTGAAGATAGGA-BHQ1	0.6		
SGLV-F	AGATTGTGCATGCTGCRTC	0.5	RdRP	172
SGLV-R	GTTTGTAAGATGCTTTCTTT	0.5		
SGLV-P	FAM-YCAAYGATGATGGTTTGACCAACCAACA-BHQ1	1		

### 3.2 Standard curves for six tick-borne pathogenic viruses

**Figure 1:**
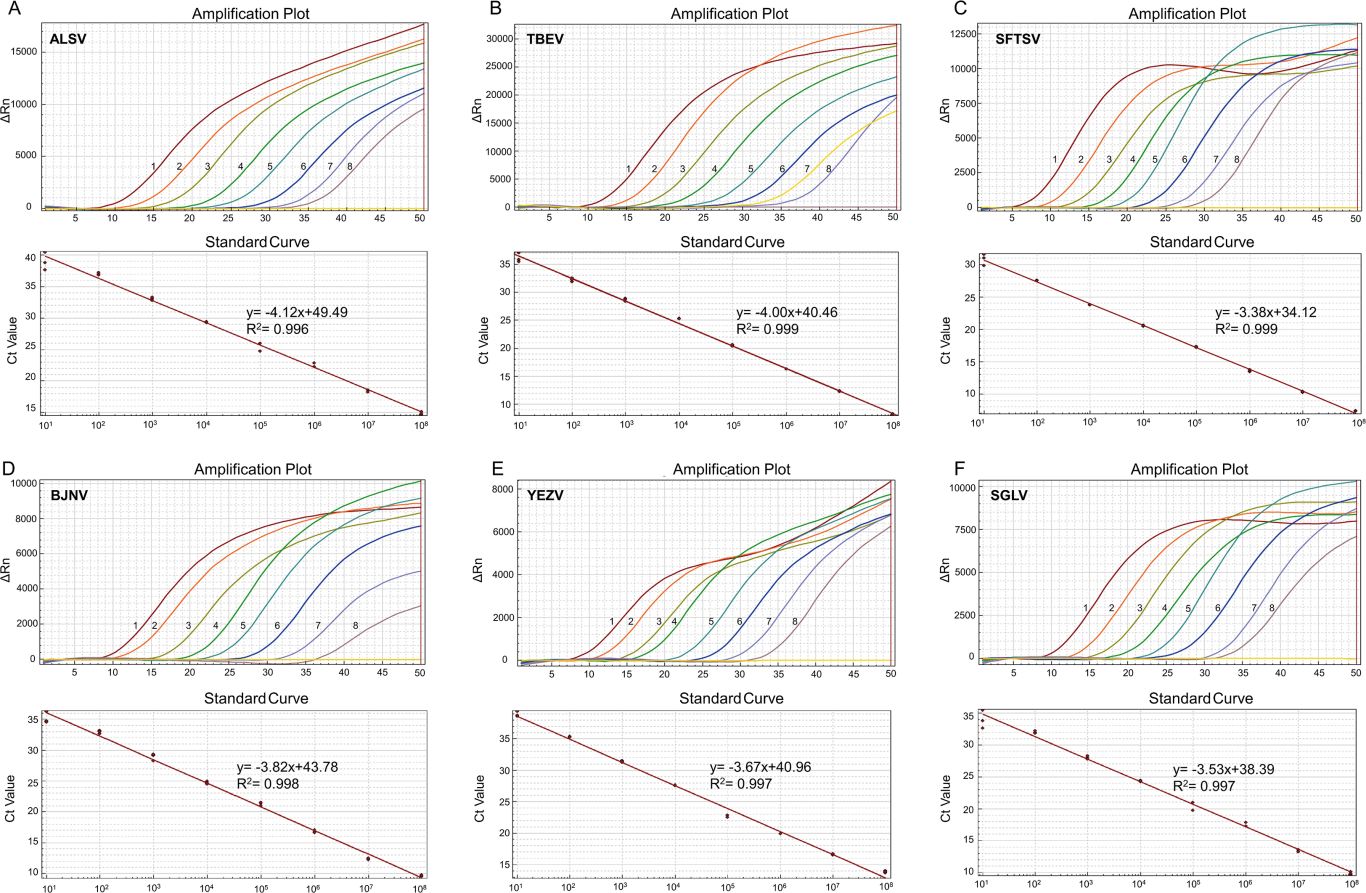
Establishment of TaqMan RT-qPCR assays for six viruses. Panels **(A–F)** show amplification plots (top) and corresponding standard curves (bottom) for ALSV, TBEV, SFTSV, BJNV, YEZV, and SGLV. Standard plasmid DNA was serially diluted from 1×10^8^ to 1×10^1^ copies/μL (Curves 1–8) to generate the plots and standard curves.

To establish standard curves, six tick-borne virus gene fragments were separately cloned into the pMD18-T vector to construct the required plasmids. The results showed the regression equations and determination coefficient (R²) as follows: Y = -4.12X + 49.49 (R²: 0.996) for ALSV; Y = -4.00X + 40.46 (R²: 0.999) for TBEV; Y = -3.38X + 34.12 (R²: 0.999) for SFTSV; Y = -3.82X + 43.78 (R²: 0.998) for BJNV; Y = -3.67X + 40.96 (R²: 0.997) for YEZV; and Y = -3.53X + 38.39 (R²: 0.997) for SGLV (**Figure 1**).

### 3.3 Sensitivity and specificity of the TaqMan RT-qPCR assays

**Figure 2:**
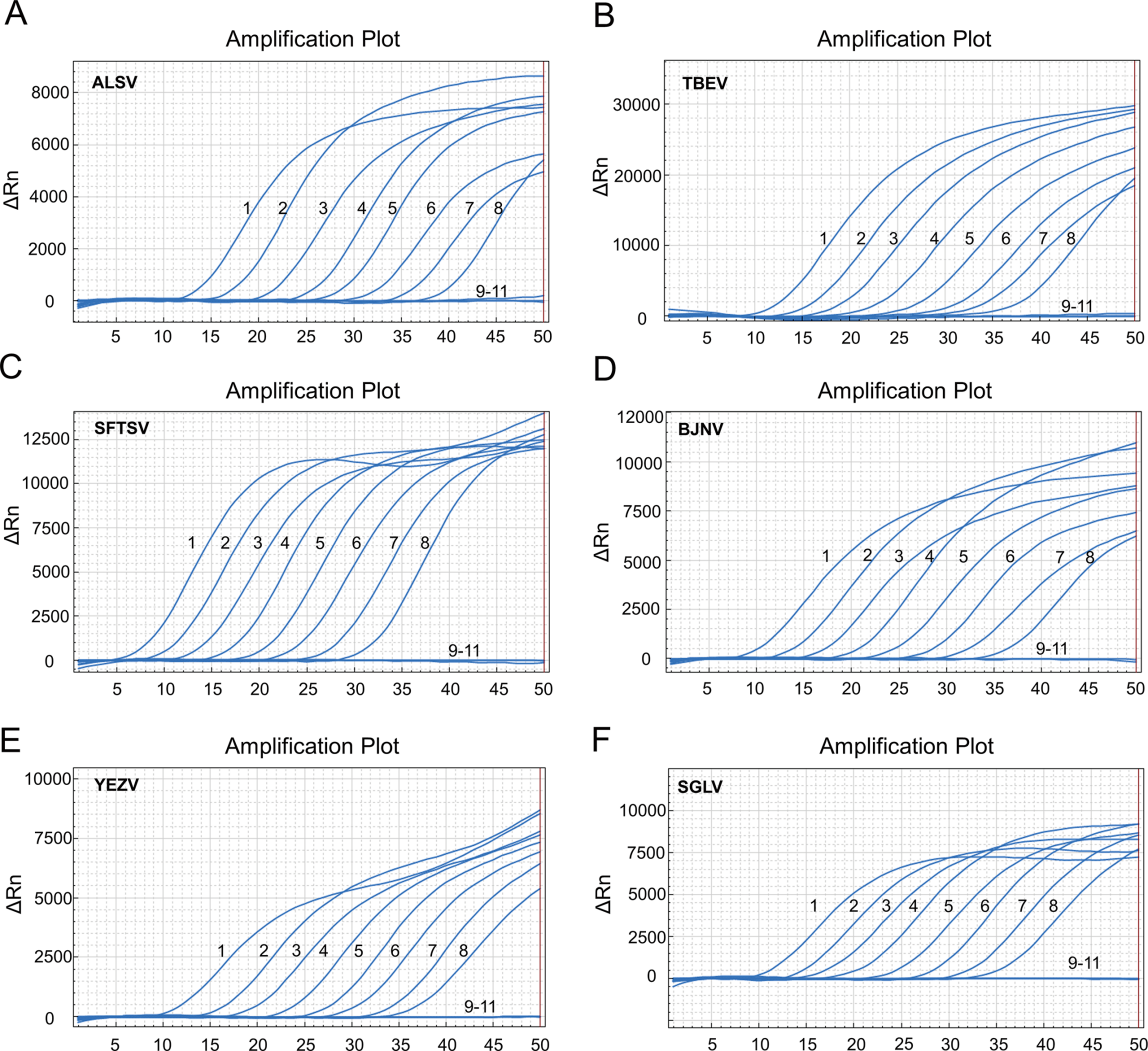
Sensitivity analysis of the established single-plex TaqMan RT-qPCR assays. Amplification plots for **(A)** ALSV, **(B)** TBEV, **(C)** SFTSV, **(D)** BJNV, **(E)** YEZV, and **(F)** SGLV. Standard plasmid DNA was serially diluted from 1×10^8^ to 1×10–^1^ copies/μL (Curves 1–10) to assess the detection limits. Curve 11, nuclease-free water as a negative control.

**Figure 3:**
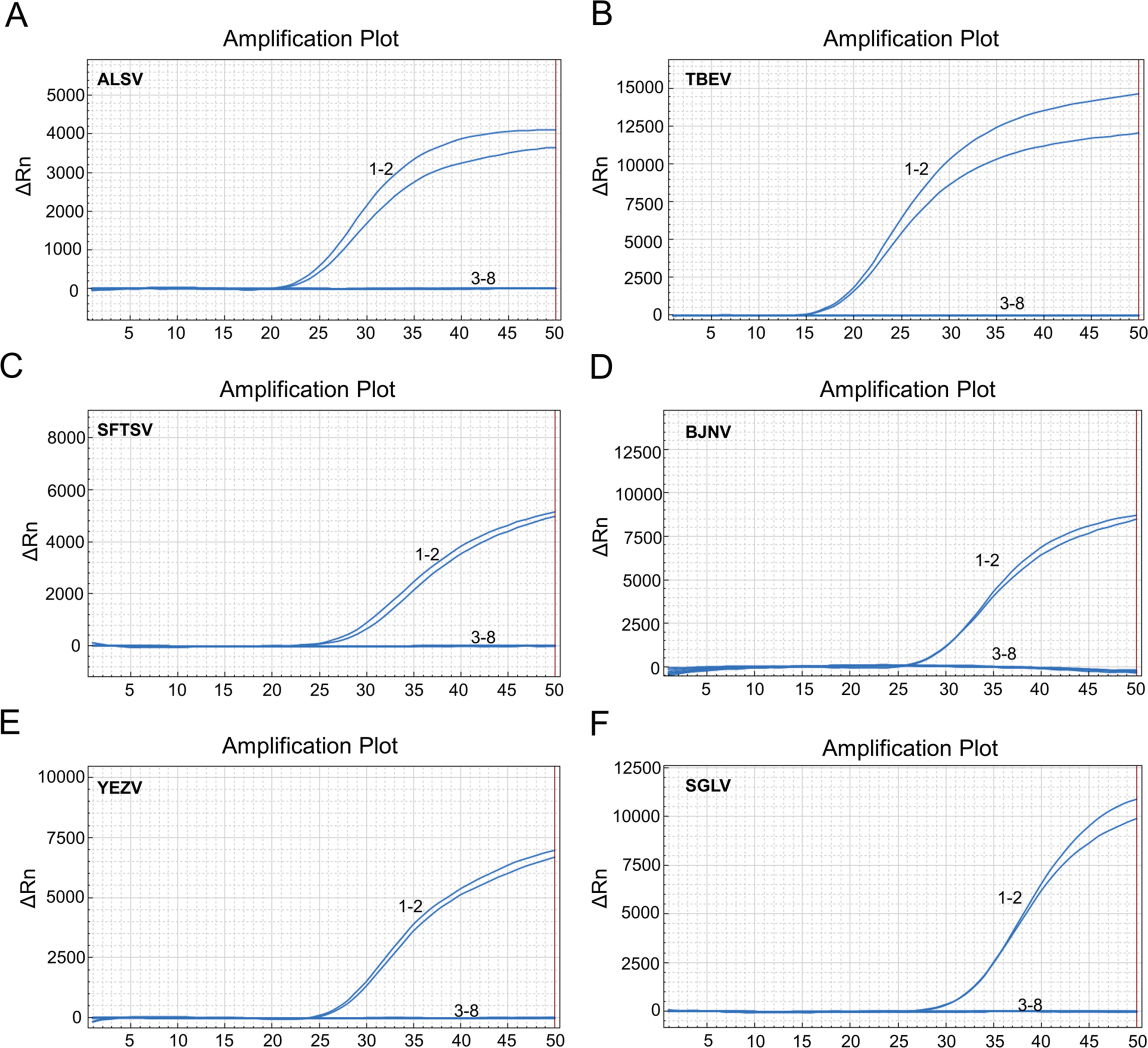
Specificity analysis of the established single-plex TaqMan RT-qPCR assays. Amplification plots for **(A)** ALSV, **(B)** TBEV, **(C)** SFTSV, **(D)** BJNV, **(E)** YEZV, and **(F)** SGLV. Curves 1–2: positive samples of the target virus; curves 3–7: cross-reactivity testing using positive samples of the other five viruses; curve 8: negative control (nuclease-free ddH_2_O).

Sensitivity analysis revealed that the detection limit was as low as 10 copies/μL for all the six viruses (**Figure 2**). Specificity analysis confirmed that no cross-reactivity occurred among the six viruses (**Figure 3**). Each virus generated positive amplification curve with its corresponding positive sample, while no amplification curves were observed for other viruses or for water.

### 3.4 Validation of the viruses in tick samples

**Figure 4:**
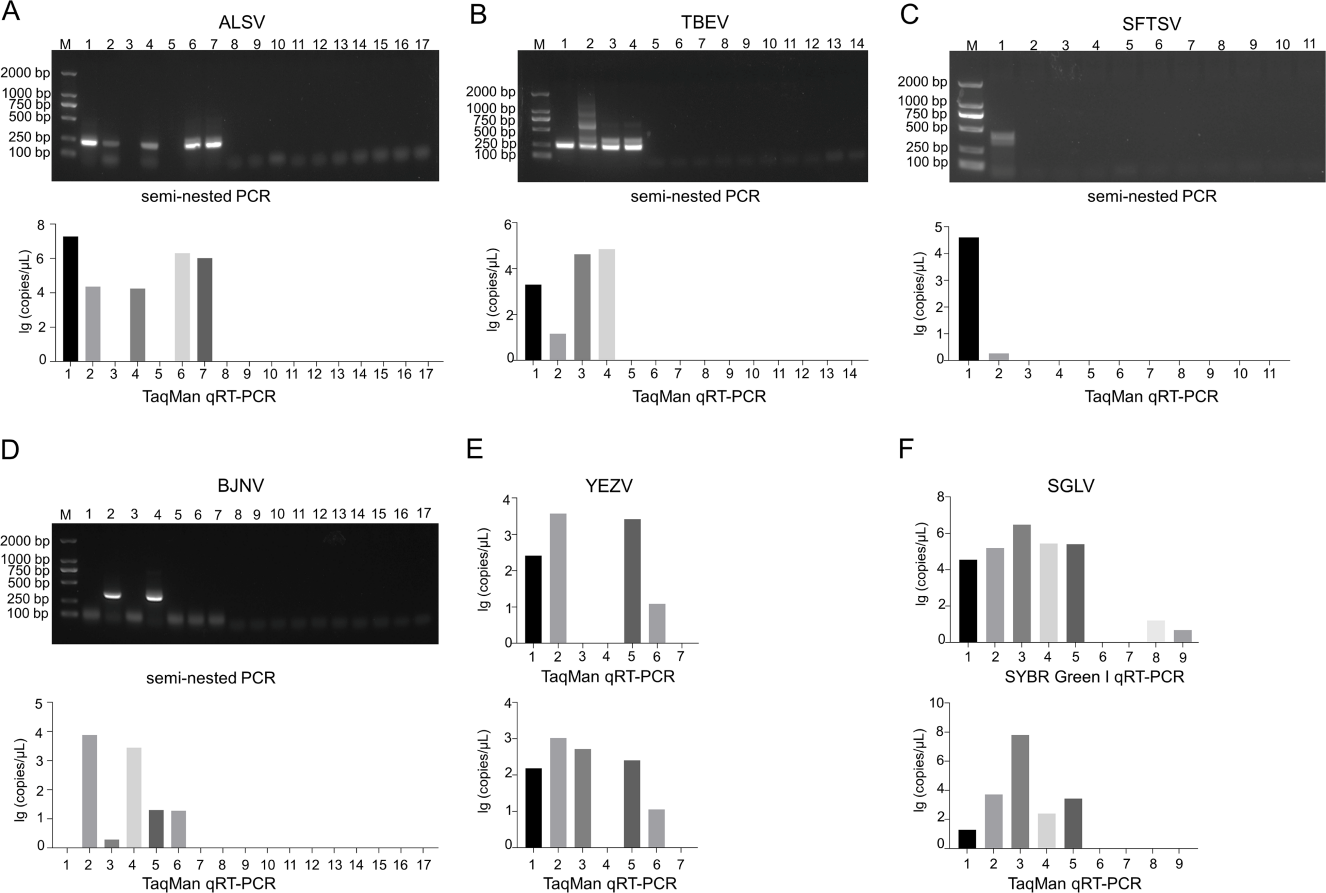
Validation of TaqMan RT-qPCR assays in field tick samples. Panels show results from established TaqMan RT-qPCR (bottom) compared to reference assays (top). Reference methods include semi-nested PCR for **(A)** ALSV, **(B)** TBEV, **(C)** SFTSV, and **(D)** BJNV; a reference TaqMan RT-qPCR for **(E)**YEZV; and SYBR Green RT-qPCR for **(F)** SGLV. Numbered lanes indicate individual tick sample pools for each virus; lanes M represent the DL2000 DNA marker.

A total of 745 adult ticks, including 89 *Haemaphysalis concinna*, 109 *Haemaphysalis concinna*, and 547 *Ixodes persulcatus* ticks, were collected from northeastern China. The collection sites were distributed in Yakeshi (n = 310) in Inner Mongolia Autonomous Region, Tahe (n = 167) in Heilongjiang Province, Wangqing (n = 159) in Jilin Province, and Fengcheng (n=109) in Liaoning Province (**Supplementary Table 2**). In total, 5/17, 4/14, 1/11, and 2/17 tick pools tested positive for ALSV, TBEV, SFTSV, and BJNV, respectively, using the reference semi-nested PCR assay. Additionally, 4/7 tick pools were positive for YEZV using the TaqMan RT-qPCR assay, and 7/9 were positive for SGLV using the SYBR Green RT-qPCR assay (**Figure 4**). The same positive tick samples for ALSV and TBEV were detected by both the established TaqMan RT-qPCR assays and the reference methods, and the viral copy number generally correlated with the intensity of the PCR bands (**Figures 4A, B**; **Supplementary Table 2**). In contrast, the established TaqMan RT-qPCR assays detected one, three, and one additional positive samples for SFTSV, BJNV, and YEZV, respectively, all of which contained low viral copy numbers that were missed by the reference methods (**Figures 4C–E**; **Supplementary Table 2**). Notably, two SGLV-positive tick sample detected by the SYBR Green RT-qPCR assay tested negative with the TaqMan RT-qPCR assay. Also, viral copy numbers in SGLV-positive pools were lower than those detected by SYBR Green RT-qPCR (**Figure 4F**). No positive amplification was observed in either the Nuclease-free ddH_2_O or the cDNA from laboratory-reared pathogen-free ticks using the six established key tick-borne TaqMan assays (**Supplementary Figure 1**).

## 4 Discussion

The widespread of emerging and re-emerging tick-borne viruses in northeastern China pose significant challenges to public health and the diagnosis of tick-borne diseases (Ziyan Liu, 2025). Since clinical symptoms of most tick-borne viral infections are non-specific, identifying the causative pathogen based solely on clinical features is challenging (Wang et al., 2019; Kodama et al., 2021; Ma et al., 2021; Wang et al., 2021). Furthermore, serological cross-reactions within the same viral family or genus complicate the differentiation of the infecting agent using single antibody detection methods. Therefore, rapid and reliable viral nucleic acid detection methods are urgently needed for both epidemiological surveillance and clinical diagnosis. In this study, we developed RT-qPCR assays for six prevalent tick-borne viruses in northeastern China, demonstrating high sensitivity and specificity. These assays offer potential for the effective monitoring and control of tick-borne diseases.

Multiplex TaqMan RT-qPCR enables simultaneous detection of six or more pathogens (van Gent-Pelzer et al., 2024). However, incorporating multiple primers and probes into a single reaction system can reduce assay sensitivity and promote non-specific amplification. This may lead to false-negative results caused by primer-primer interactions and the competitive consumption of reaction components. Furthermore, relying heavily on the limited detection channels of an instrument can constrain assay scalability and flexibility, often necessitating high-end platforms. Given these limitations, we adopted a single-plex fluorescence quantitative PCR approach. Although this method requires more cDNA input and reduces detection throughput, it provides enhanced sensitivity and specificity, which justifies its application.

Current methods for detecting the nucleic acids of these six tick-borne viruses include semi-nested PCR, SYBR Green RT-qPCR, TaqMan RT-qPCR, and loop-mediated isothermal amplification (LAMP) (Zeng et al., 2017; Wang et al., 2019; Kodama et al., 2021; Li et al., 2022; Gui et al., 2024; Huang et al., 2025; Wang et al., 2025; Opota et al., 2026). However, a lack of standardization hinders epidemiological surveillance and reduces testing efficiency. Semi-nested PCR is cost-effective but prone to non-specific amplification; it requires confirmation via Sanger sequencing, which prolongs detection time and limits overall sensitivity. SYBR Green RT-qPCR is also economical but lacks probe-based specificity, often leading to false positives, particularly in samples with low viral loads. Although TaqMan RT-qPCR assays have been developed for many of these viruses, established methods usually do not include enough viral strains originating from Northeast China, which can lead to the missed detection of positive samples. Furthermore, while LAMP assays enable visual detection, they are unable to perform quantitative analysis. Given these limitations, this study extensively collected viral sequences from Northeast China to design specific primers and establish a targeted TaqMan RT-qPCR assay, making it highly suitable for rapid, localized detection in this region.

This study has several limitations. First, certain tick-borne viruses prevalent in northeastern China, such as Jingmen tick virus, Xuecheng virus, and Wetland virus, were not included due to the lack of positive tick samples for these pathogens. Additionally, human infection samples were unavailable for these viruses, preventing validation of the established method in human specimens. Therefore, the method is currently suitable for epidemiological screening in ticks, but its application to human samples requires further verification.

## 5 Conclusion

Our single-plex TaqMan RT-qPCR assays provides a highly sensitive and specific method for detecting emerging and re-emerging tick-borne viruses circulating in northeastern China. The establishment of these assays facilitates rapid viral detection in ticks and offers a potentially reliable diagnostic tool for clinical applications, thereby strengthening prevention and control efforts against tick-borne viral diseases in China.

## Data Availability

The original contributions presented in the study are included in the article/**Supplementary Material**. Further inquiries can be directed to the corresponding authors.

## Supplementary material

Supplementary Figure 1 No positive amplification was observed in the reagent controls using the six established key tick-borne TaqMan assays. Curves 1–6, nuclease-free ddH_2_O; 7–12, cDNA from laboratory-reared pathogen-free ticks.
