# Supplementary figures and images for: Development and evaluation of single-plex TaqMan real-time quantitative PCR assays for the detection of six tick-borne pathogenic viruses in northeastern China
Source: Front Cell Infect Microbiol. 2026 Jun 4;16:1777543. doi: 10.3389/fcimb.2026.1777543 (PMC13275643; doi:10.3389/fcimb.2026.1777543)

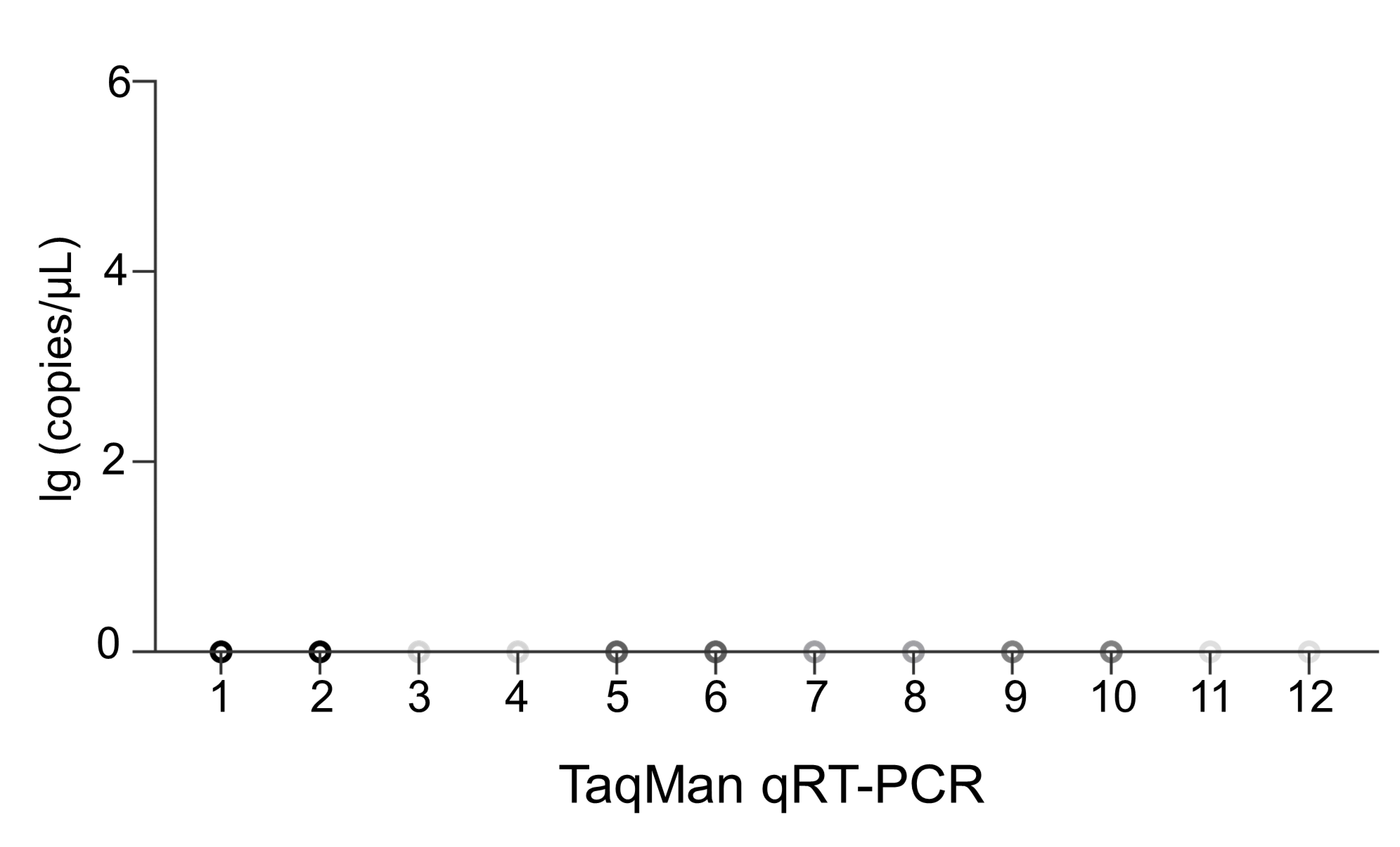

Supplement: Supplementary Figure 1 — No positive amplification was observed in the reagent controls using the six established key tick-borne TaqMan assays. Curves 1–6, nuclease-free ddH2O; 7–12, cDNA from laboratory-reared pathogen-free ticks. [file Image1.tif]
