# Supplementary material for: Development and evaluation of single-plex TaqMan real-time quantitative PCR assays for the detection of six tick-borne pathogenic viruses in northeastern China
Source: Front Cell Infect Microbiol. 2026 Jun 4;16:1777543. doi: 10.3389/fcimb.2026.1777543 (PMC13275643; doi:10.3389/fcimb.2026.1777543)
Supplement: Supplementary file 2 [file Table1.docx]

**Supplementary Appendix**

[**Table S1** Nucleotide sequences of six tick-borne viruses utilized for the design of specific primers and probes. 2](#_Toc228617287)

[**Table S2.** Detection of six tick-borne viruses by TaqMan RT-qPCR in field-collected ticks, northeastern China. 5](#_Toc228617288)

# **Table S1** Nucleotide sequences of six tick-borne viruses utilized for the design of specific primers and probes.

| **Virus** | **GenBank No.** | **Strains** |
| --- | --- | --- |
| ALSV | OP921097 | GR RM/Switzerland/2022 |
|  | ON408068 | NE-TH4 |
|  | MW525319 | Miass506 |
|  | MW525315 | Miass502 |
|  | MW525313 | Tat14-T21924 |
|  | MW525312 | Ulya15-T22688 |
|  | MW525310 | Rowan19-T32778 |
|  | MW525309 | Kursh18-T30290 |
|  | MW525308 | Kursh18-T30286 |
|  | MW525307 | Kursh18-T30285 |
|  | MW525306 | Kursh18-T30284 |
|  | MW525305 | Kursh18-T30281 |
|  | MW525304 | Kursh18-T30280 |
|  | MW525303 | Kursh18-T30278 |
|  | MW525301 | Kursh18-T27123 |
|  | MW525300 | Kursh17-T25652 |
|  | MW525287 | Goms13-T17158 |
|  | MT536951 | HLJ2 |
|  | MT536950 | HLJ1 |
|  | MN648776 | Miass519 |
|  | MN648772 | Miass527 |
|  | MN604229 | Galozero-14-T20426 |
|  | MN107158 | Haapasaari-18 |
|  | MN107154 | Kuutsalo-23 |
|  |  |  |
| TBEV | MT246197 | JLJiaohepolyproteingene |
|  | OP037818 | RSSEV |
|  | MT710343 | 234polyproteingene |
|  | MT710341 | 506 |
|  | MT710340 | G67-20 |
|  | MT681748 | 62199 |
|  | MT681747 | 66113 |
|  | MT671302 | ChB |
|  | MT671301 | 1024 |
|  | MT671300 | 95 |
|  | MN115817 | 1020-69 |
|  | LC440459 | Sapporo-17-Io1 |
|  | JQ825147 | Oshima5.10 |
|  | KU761576 | Sofjin-1953 |
|  | KU761575 | Primorye-1285 |
|  | KU761573 | Primorye-1056 |
|  | KU761572 | Primorye-1035 |
|  | KT069219 | Primorye-1001 |
|  | KU761570 | Primorye-949 |
|  | KU761569 | Primorye-696 |
|  | KT069219 | Primorye-1001 |
|  |  |  |
| SFTSV | MT309101 | L |
|  | MT309100 | L |
|  | MT309099 | L |
|  | KF887445 | LN2012-58 |
|  | KF887444 | LN2012-42 |
|  | KF887443 | LN2012-41 |
|  | KF887442 | LN2012-34 |
|  | KF887441 | LN2012-14 |
|  | MT232962 | LNHDG2019-6 |
|  | MF357030 | DanDong292 |
|  | MF357029 | DanDong273 |
|  | MF357028 | DanDong203 |
|  |  |  |
| BJNV | NC 079010 | H160 |
|  | MW315112 | H1063 |
|  | MW315111 | H56 |
|  | MW315110 | H59 |
|  | MW315109 | H39 |
|  | MW315108 | H801 |
|  | MW315107 | H160 |
|  | MN122100 | EEGN25 |
|  | MN122098 | SW41 |
|  | MN122096 | ALS66 |
|  | MN122094 | YC846 |
|  | MN122092 | YC908 |
|  | MN122090 | DH73 |
|  | MN122088 | YC854 |
|  | MN122086 | DH58 |
|  | MN122084 | YKS322 |
|  | MN122082 | YKS59 |
|  | MN122080 | YKS44 |
|  | ON408107 | NE-DH3 |
|  | ON408105 | NE-YC3 |
|  | ON408103 | NE-YC4 |
|  | ON408101 | NE-TH4 |
|  | ON408099 | NE-TH3 |
|  | ON408097 | NE-SL3 |
|  | ON408095 | NE-SL4 |
|  |  |  |
| YEZV | NC 079098 | HH003-2020 |
|  | LC735735 | BT-1864 |
|  | LC735732 | BT-1844 |
|  | LC735729 | BT-1826 |
|  | LC735726 | BT-1821 |
|  | ON563284 | H-IM01 |
|  | ON563281 | T-JL01 |
|  | ON563278 | T-IM01 |
|  | ON563275 | T-HLJ03 |
|  | ON563272 | T-HLJ02 |
|  | ON563269 | T-HLJ01 |
|  | LC621359 | HH011-2020 |
|  | LC621356 | HH003-2020 |
|  |  |  |
| SGLV | MT328773 | JA86 |
|  | MT328770 | HLJ1175 |
|  | MT328767 | HLB178 |
|  | MT328764 | GH1185 |
|  | MT328776 | HLJ1202 |
|  | ON811868 | TIGMIC3 |
|  | ON811866 | TIGMIC2 |
|  | ON811835 | TIGMIC1 |
|  | ON408079 | NE-TH2 |
|  | ON408076 | NE-TH1 |
|  | MT328779 | YC585 |
|  | MT328776 | HLJ1202 |

# **Table S2.** Detection of six tick-borne viruses by TaqMan RT-qPCR in field-collected ticks, northeastern China.

| Province | Location | No. of positive pools/ no. of tick pools | | | | | |
| --- | --- | --- | --- | --- | --- | --- | --- |
|  |  | *Ixodes persulcatus* | | | | *Haemaphysalis longicornis* | *Haemaphysalis concinna* |
|  |  | ALSV | TBEV | YEZV | BJNV | SFTSV | SGLV |
| Inner Mongolia | Yakeshi | nd | 4/14 | nd | 5/17 | nd | nd |
| Heilongjiang | Tahe | 5/17 | nd | nd | nd | nd | nd |
| Jilin | Wangqing | nd | nd | 5/7 | nd | nd | 5/9 |
| Liaoning | Fengcheng | nd | nd | nd | nd | 2/11 | nd |
| Total | | 5/17 | 4/14 | 5/7 | 5/17 | 2/11 | 5/9 |

*nd, no data.
